# Hepatotoxicity of statins: a real-world study based on the US Food and Drug Administration Adverse Event Reporting System database

**DOI:** 10.3389/fphar.2024.1502791

**Published:** 2025-01-07

**Authors:** Bojing Wang, Shu Huang, Shiqi Li, Yaqi Deng, Ziyan Li, Yizhou Wang, Xiaomin Shi, Wei Zhang, Lei Shi, Xiaohong Wang, Xiaowei Tang

**Affiliations:** ^1^ Department of Gastroenterology, The Affiliated Hospital of Southwest Medical University, Luzhou, China; ^2^ Department of Gastroenterology, Lianshui County People’ Hospital, Huaian, China; ^3^ Department of Gastroenterology, Lianshui People’ Hospital of Kangda College Affiliated to Nanjing Medical University, Huaian, China; ^4^ Department of Gastroenterology, Xuzhou Central Hospital, Xuzhou Clinical School of Xuzhou Medical University, Xuzhou, China

**Keywords:** statins, statin drugs, FAERS, hepatotoxicity, hepatic disorder, adverse event

## Abstract

**Background:**

Statins, as an important class of lipid-lowering drugs, play a key role in the prevention and treatment of cardiovascular diseases. However, with their widespread use in clinical practice, some adverse events have gradually emerged. In particular, the hepatotoxicity associated with statins use has become one of the clinical concerns that require sufficient attention.

**Methods:**

In this study, we conducted a comprehensive and detailed analysis of the hepatotoxicity of statins based on the data of the US Food and Drug Administration Adverse Event Reporting System database from the first quarter (Q1) of 2004 to the Q1 of 2024 and used Reporting Odds Ratios and Empirical Bayes Geometric Mean to mine the signal of adverse events.

**Results:**

In this study, hepatic disorder related seven statins all exhibited positive signals. Through signal mining, we identified a total of 14,511 cases of adverse events associated with hepatic disorder caused by these statin drugs, with atorvastatin, simvastatin, and rosuvastatin occurring at a higher rate. A total of 148 positive signals related to adverse events of hepatic disorder were captured. Autoimmune hepatitis and drug-induced liver injury both presented positive signals across multiple statin drugs. Notably, atorvastatin had the most significant signal strength in cholestatic pruritus and bilirubin conjugation abnormal. Fluvastatin also showed notable signal strength in autoimmune hepatitis, while simvastatin had a relatively weaker signal strength for hepatic enzyme increased.

**Conclusion:**

This study discovered specific adverse event signal values, revealing potential hepatotoxic risks associated with the use of statin drugs. The results provide an important reference for the safe clinical use of drugs, help to improve the understanding of the safety of statins, and also provide a scientific basis for clinicians to make more accurate and safe decisions when making treatment plans.

## Introduction

Statins, a primary class of lipid-lowering drugs, occupy an irreplaceable position in the field of cardiovascular medicine (Vinci et al., 2021; Cardiovascular disease, 2023). This class of drugs blocks the synthesis of cholesterol in the liver by specifically inhibiting the activity of a key enzyme, 3-hydroxy-3-methylglutaryl coenzyme A (HMG-CoA) reductase (Sirtori, 2014; Liu et al., 2019). This mechanism not only significantly reduces levels of total cholesterol (total-C), low-density lipoprotein cholesterol (LDL-C), apolipoprotein B (apoB) and triglycerides (TG) in the blood (Liu et al., 2019; Oesterle et al., 2017), but also offers long-term benefits in promoting vascular health and preventing atherosclerosis (Cai et al., 2021).

As the incidence of cardiovascular diseases continues to rise globally, the clinical demand for statins is also increasing. It is reported that the market share of statin drugs worldwide is continuously expanding, even formulating a trend of “statinization” globally (Blais et al., 2021). The commonly used statin drugs in clinical practice include atorvastatin, fluvastatin, lovastatin, pitavastatin, pravastatin, rosuvastatin, and simvastatin. Cerivastatin was withdrawn from the market in 2001 due to the serious adverse event of rhabdomyolysis. Correspondingly, adverse events associated with statin use have been continuously reported, including statin-associated muscle symptoms (SAMS) (Vinci et al., 2021; Thompson et al., 2016), diabetes mellitus (DM) (Thompson et al., 2016), and neurocognitive disorders (Xiao et al., 2024).

Active liver disease is a contraindications for the use of statins. Currently, numerous studies have analyzed the influence of statins on liver-related diseases, with some suggesting that statin use is significantly associated with liver disease protection and may be beneficial in the treatment of liver diseases (Vell et al., 2023). However, Liang X et al.’s study clearly indicated that statins increase the risk of liver damage (Liang et al., 2018). Therefore, it is very necessary to conduct further analysis of the hepatotoxicity of statins in this study.

The purpose of this study was to conduct an in-depth analysis of real-world data from the US Food and Drug Administration Adverse Event Reporting System (FAERS) database to investigate hepatic disorders caused by statin drugs, thereby contributing to a deeper understanding of their safety. In addition, the results of the study will provide certain reference information for clinical drug use, and help clinicians better weigh the benefits and risks of statins, thereby further optimizing treatment plans and maximizing the therapeutic effect.

## Methods

### Data source

The FAERS database is a widely recognized and extensive public resource dedicated to drug related safety information. We extracted all data on statins from the FAERS database spanning from the first quarter (Q1) of 2004 to the first quarter (Q1) of 2024. The following seven statins were included in this study: atorvastatin, fluvastatin, lovastatin, pitavastatin, pravastatin, rosuvastatin and simvastatin.

**FIGURE 1:**
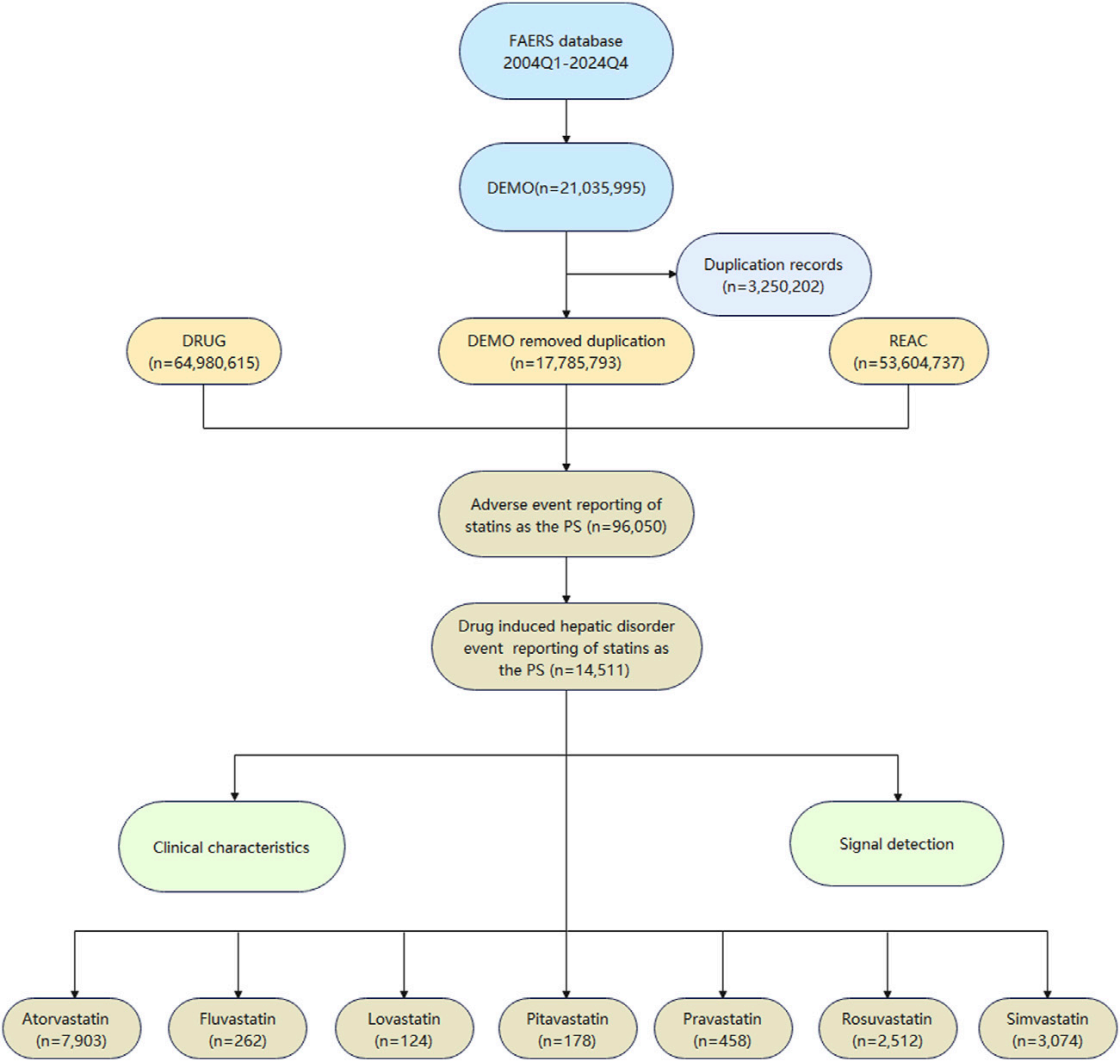
The flowchart of data extraction from the FAERS database.

Following the flow chart presented in Figure 1, we meticulously processed the data. Initially, we obtained 21,035,995 case records. After removing duplicates, we filtered out 17,785,793 unique case records. Focusing on statins as the drugs of Primary Suspect (PS), we identified 96,050 statins-related adverse event reports, with a total of 14,511 cases involving drug-related hepatic disorder.

### Drug related hepatic disorder event determination

In the FAERS database, all recorded adverse event terms are standardized by MedDRA (Medical Dictionary for Regulatory Activities). This study initially utilized standardized MedDRA analysis queries (SMQ) [Code: 2000006] to conduct a broad search on “Drug related hepatic disorders-comprehensive search,” preliminarily identifying 376 Preferred Terms (PTs). Subsequently, a more narrow search was conducted, further selecting 304 PTs. After deduplication, a final set of 244 PTs related to drug related hepatic disorder events was established. The PT set was shown in Supplementary Table S1.

### Signal mining and statistical analysis

In this study, we employed two methods, the Reporting Odds Ratio (ROR) and the Empirical Bayes Geometric Mean (EBGM), to mine and analyze the signals of adverse events associated with statins. According to the established criteria, a greater than 3 and lower limit of 95% confidence interval of ROR value is greater than 1, indicating a positive signal; for EBGM, a lower limit of the 95% confidence interval greater than 2 is also regarded as a positive signal. The specific calculation method of ROR EBGM are shown in Supplementary Tables S2, S3. Higher values of ROR and EBGM indicate stronger signals, which suggest a stronger association between the target drug and the target adverse event. This study primarily used R software version 4.4.0 and Microsoft Excel for statistical analysis.

## Results

### Clinical characteristics distribution of reported case

In this study, we identified a total of 14,511 cases of hepatic disorder adverse events related to the use of statins. The number of atorvastatin-related cases was the highest at 7,903, followed by simvastatin (n = 3,074) and rosuvastatin (n = 2,512). In comparison, there were fewer cases of hepatic disorder caused by fluvastatin, lovastatin, pitavastatin, and pravastatin as shown in Figure 1.

Table 1 illustrated the clinical characteristics of statins in drug-related hepatic disorder. There were no significant differences in adverse events with statins between females and males (4,644, 46% vs. 4,699, 46.5%). At the age level, most of adverse events were concentrated in people aged 65–85 years (4,358, 43.2%), with rosuvastatin showing nearly identical incidence rates of adverse events in people aged 18–65 years and 65–85 years. Regarding the reporters, physicians undertook the majority of reporting, accounted for the majority of the reports (3470, 34.4%). Serious outcomes of statins adverse events occurred more frequently in hospitalization-initial or prolonged and other important medical events. Overall, the occurrence of fatal outcomes associated with statins was relatively low, accounting for 14% of the total. Simvastatin had the highest fatality rate (585, 19%), followed by fluvastatin (39, 15%) and lovastatin (19, 15%), while pitavastatin had the lowest proportion, at only 8%.

**TABLE 1:** Clinical characteristics distribution of hepatic disorder caused by seven statins included in this study.

	Atorvastatin	Fluvastatin	Lovastatin	Pitavastatin	Pravastatin	Rosuvastatin	Simvastatin	Statins
	(n = 5,447)	(n = 170)	(n = 78)	(n = 116)	(n = 333)	(n = 1813)	(n = 2,139)	(n = 10,096)
Gender								
Female	2,652 (48.7%)	89 (52.4%)	27 (34.6%)	44 (37.9%)	151 (45.3%)	809 (44.6%)	872 (40.8%)	4,644 (46.0%)
Male	2,409 (44.2%)	67 (39.4%)	48 (61.5%)	35 (30.2%)	147 (44.1%)	916 (50.5%)	1,077 (50.4%)	4,699 (46.5%)
Unknown	386 (7.1%)	14 (8.2%)	3 (3.8%)	37 (31.9%)	35 (10.5%)	88 (4.9%)	190 (8.9%)	753 (7.5%)
Age								
<18	14 (0.3%)	1 (0.6%)	2 (2.6%)	0 (0%)	2 (0.6%)	19 (1.0%)	4 (0.2%)	42 (0.4%)
18∼64.9	1,821 (33.4%)	66 (38.8%)	23 (29.5%)	32 (27.6%)	119 (35.7%)	599 (33.0%)	692 (32.4%)	3,352 (33.2%)
65∼85	2,512 (46.1%)	71 (41.8%)	30 (38.5%)	34 (29.3%)	139 (41.7%)	608 (33.5%)	964 (45.1%)	4,358 (43.2%)
>85	363 (6.7%)	9 (5.3%)	1 (1.3%)	3 (2.6%)	14 (4.2%)	62 (3.4%)	126 (5.9%)	578 (5.7%)
Unknown	737 (13.5%)	23 (13.5%)	22 (28.2%)	47 (40.5%)	59 (17.7%)	525 (29.0%)	353 (16.5%)	1,766 (17.5%)
Reporter								
Consumer	958 (17.6%)	15 (8.8%)	14 (17.9%)	11 (9.5%)	59 (17.7%)	258 (14.2%)	280 (13.1%)	1,595 (15.8%)
Health Professionals	669 (12.3%)	51 (30.0%)	0 (0%)	11 (9.5%)	23 (6.9%)	362 (20.0%)	118 (5.5%)	1,234 (12.2%)
Lower	41 (0.8%)		0 (0%)	1 (0.9%)	0 (0%)	0 (0%)	1 (0.0%)	43 (0.4%)
Physician	1,880 (34.5%)	31 (18.2%)	28 (35.9%)	60 (51.7%)	99 (29.7%)	778 (42.9%)	594 (27.8%)	3,470 (34.4%)
Other health professionals	1,087 (20.0%)	56 (32.9%)	12 (15.4%)	17 (14.7%)	79 (23.7%)	165 (9.1%)	611 (28.6%)	2,027 (20.1%)
Pharmacist	729 (13.4%)	7 (4.1%)	16 (20.5%)	5 (4.3%)	55 (16.5%)	155 (8.5%)	339 (15.8%)	1,306 (12.9%)
Registered nurse	1 (0.0%)		0 (0%)			0 (0%)	0 (0%)	1 (0.0%)
Unknown	82 (1.5%)	10 (5.9%)	8 (10.3%)	11 (9.5%)	18 (5.4%)	95 (5.2%)	196 (9.2%)	420 (4.2%)
Serious outcome								
Death	297 (4.1%)	9 (4.1%)	4 (4.1%)	2 (1.5%)	14 (3.1%)	62 (2.6%)	170 (5.6%)	558 (4.1%)
Disability	199 (2.7%)	25 (11.4%)	9 (9.2%)	4 (3.0%)	14 (3.1%)	74 (3.1%)	205 (6.7%)	530 (3.9%)
Hospitalization-initial or prolonged	2,721 (37.3%)	67 (30.5%)	34 (34.7%)	41 (31.1%)	152 (34.2%)	777 (32.4%)	1,228 (40.1%)	5,020 (36.8%)
Life-threatening	359 (4.9%)	10 (4.5%)	6 (6.1%)	8 (6.1%)	21 (4.7%)	155 (6.5%)	207 (6.8%)	766 (5.6%)
Other important medical events	3,250 (44.6%)	92 (41.8%)	31 (31.6%)	52 (39.4%)	211 (47.4%)	1,071 (44.7%)	1,067 (34.9%)	5,774 (42.3%)
Congenital anomaly	1 (0.0%)	1 (0.5%)	0 (0%)		0 (0%)	1 (0.0%)	0 (0%)	3 (0.0%)
Required intervention	41 (0.6%)	2 (0.9%)	5 (5.1%)	0 (0%)	7 (1.6%)	23 (1.0%)	73 (2.4%)	151 (1.1%)
Unknown	423 (5.8%)	14 (6.4%)	9 (9.2%)	25 (18.9%)	26 (5.8%)	235 (9.8%)	111 (3.6%)	843 (6.2%)
Fatal or non-fatal outcome								
Fatal outcome	934 (12%)	39 (15%)	19 (15%)	14 (8%)	48 (10%)	325 (13%)	585 (19%)	1,964 (14%)
Non-fatal outcome	6,969 (88%)	223 (85%)	105 (85%)	164 (92%)	410 (90%)	2,187 (87%)	2,489 (81%)	12,547 (86%)

**FIGURE 2:**
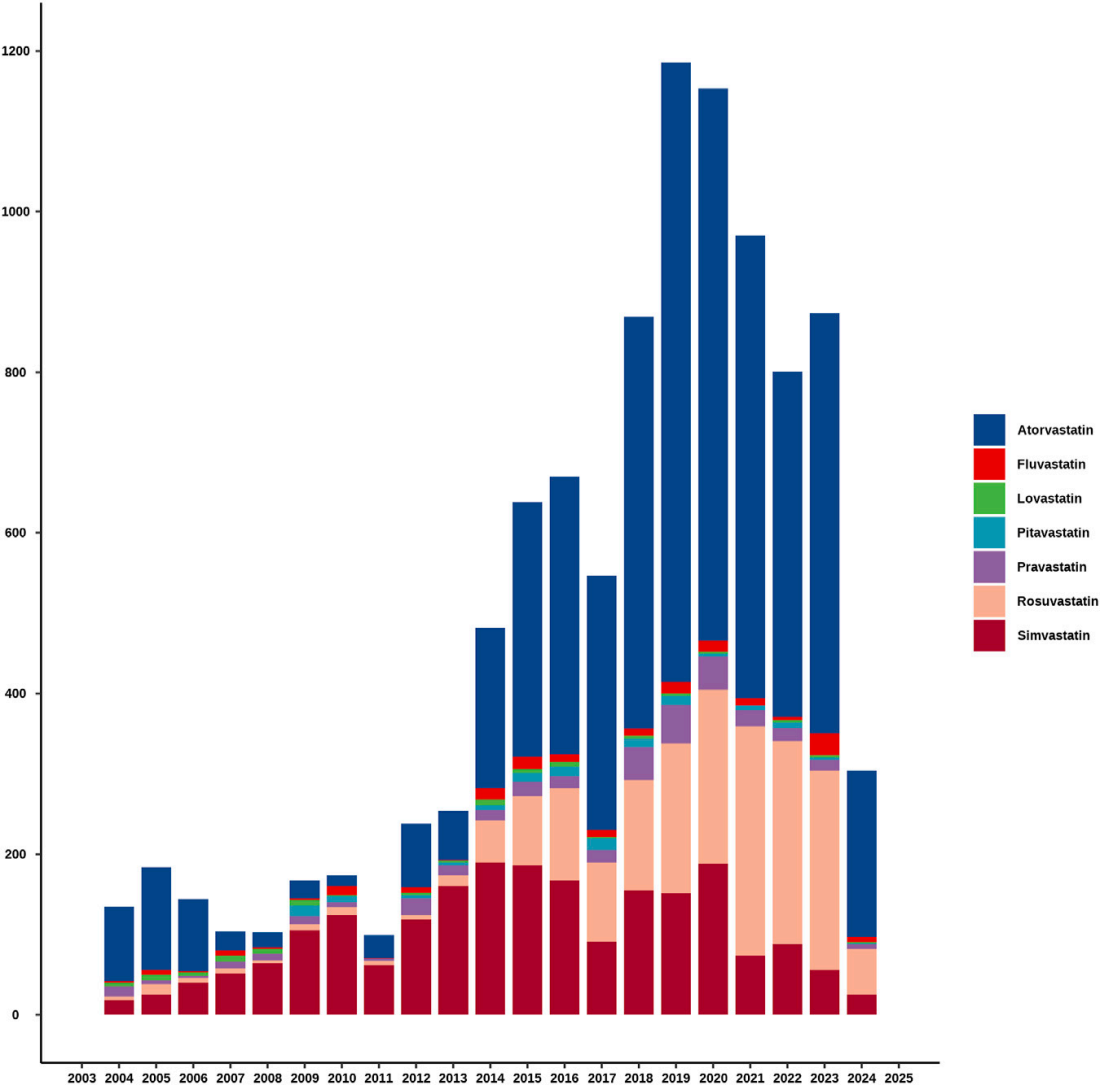
The annual reported cases of statins-related hepatic disorder during the study period.

Figure 2 clearly showed the trend in the number of adverse event reports of hepatic disorders caused by statin drugs over the years. Overall, the number of reported cases exhibited an increasing trend year by year. Notably, between 2018 and 2023, the number of reported cases each year exceeded 800. During the research period, the highest number of reports was in 2019, with 1,186 cases, accounting for 11.7% of the total. In terms of specific drugs, atorvastatin accounted for the highest number of hepatic disorder cases, followed by simvastatin and rosuvastatin, with the specific reported numbers shown in Table 2.

**TABLE 2:** Specific data on cases of statin-related hepatic disorder reported during the study period.

	Atorvastatin	Fluvastatin	Lovastatin	Pitavastatin	Pravastatin	Rosuvastatin	Simvastatin	Total
2004	93	2	5		12	5	18	135 (1.3%)
2005	128	6	7		5	13	25	184 (1.8%)
2006	90	1	5		2	6	40	144 (1.4%)
2007	24	6	8		8	7	51	104 (1.0%)
2008	19	2	6		8	4	64	103 (1.0%)
2009	22	2	7	13	10	8	105	167 (1.7%)
2010	14	11	1	8	6	10	124	174 (1.7%)
2011	28	1	0		3	5	62	99 (1.0%)
2012	79	7	3	4	21	5	119	238 (2.4%)
2013	61	1	2	4	12	14	160	254 (2.5%)
2014	200	14	7	6	13	52	190	482 (4.8%)
2015	317	15	5	11	18	86	186	638 (6.3%)
2016	346	9	6	12	15	115	167	670 (6.6%)
2017	317	9	1	15	15	99	91	547 (5.4%)
2018	513	9	3	11	41	137	155	869 (8.6%)
2019	772	14	3	11	48	187	151	1,186 (11.7%)
2020	687	14	2	4	41	217	188	1,153 (11.4%)
2021	576	9	0	6	20	285	74	970 (9.6%)
2022	430	4	3	7	16	253	88	801 (7.9%)
2023	524	27	3	3	13	248	56	874 (8.7%)
2024	207	7	1	1	6	57	25	304 (3.0%)

### Drug induced liver injury signal detection

This study employed the ROR and EBGM methods to detect and analyze signals related to statin drugs. Among the adverse events associated with statin drugs, the number of drug-related hepatic disorder events and other adverse events were 14,511 and 303,623, respectively. All seven statins included in this study exhibited positive signals, with fluvastatin showing the strongest signal [ROR 8.29, 95%CI (7.29–9.42), EBGM 2.21, EBGM05 2.05] and pravastatin showing the weakest signal [ROR 2.25, 95% CI (2.05–2.47), EBGM 2.21, EBGM05 2.05]. Additionally, atorvastatin, simvastatin, and rosuvastatin had the highest frequency of drug-related hepatic disorder adverse events, with occurrences of 7,903, 3,074, and 2,512, respectively, as shown in Table 3.

**TABLE 3:** Signal mining of statins-related hepatic disorder or non-related hepatic disorder.

	Drug-related hepatic disorder				Drug non-related hepatic disorder			
	Statins-related hepatic disorder adverse events	ROR (95% CI)	EBGM	EBGM05	Statins non-related hepatic disorder adverse events	ROR (95% CI)	EBGM	EBGM05
Atorvastatin	7,903	3.94 (3.85–4.03)	3.75	3.68	141,316	0.25 (0.25–0.26)	0.96	0.94
Fluvastatin	262	8.29 (7.29–9.42)	7.51	6.75	2,208	0.12 (0.11–0.14)	0.91	0.81
Lovastatin	124	3.39 (2.83–4.06)	3.28	2.82	2,555	0.3 (0.25–0.35)	0.97	0.83
Pitavastatin	178	3.34 (2.87–3.88)	3.23	2.85	3,721	0.3 (0.26–0.35)	0.97	0.85
Pravastatin	458	2.25 (2.05–2.47)	2.21	2.05	14,192	0.44 (0.4–0.49)	0.98	0.91
Rosuvastatin	2,512	2.7 (2.6–2.81)	2.63	2.55	65,023	0.37 (0.36–0.39)	0.98	0.94
Simvastatin	3,074	2.88 (2.78–2.99)	2.8	2.72	74,608	0.35 (0.33–0.36)	0.97	0.95
Total	14,511	3.38 (3.33–3.44)	3.23	3.19	303,623	0.3 (0.29–0.3)	0.97	0.95

We identified a total of 148 preferred terms (PT) associated with drug-related hepatic disorder events across seven statin drugs, with atorvastatin having the highest number of PTs and pivastatin the fewest. In this study, among the seven statin drugs included, atorvastatin had the highest frequency of adverse events related to hepatic disorder, specifically drug-induced liver injury (n = 521), alanine aminotransferase increased (n = 500), hepatic enzyme increased (n = 458), liver function test abnormal (n = 429), and aspartate aminotransferase increased (n = 406). Autoimmune hepatitis was a common adverse event associated with all seven statin drugs, and drug-induced liver injury was observed in six of the statin drugs. Adverse events that occurred with five statin drugs include: alanine aminotransferase increased, aspartate aminotransferase increased, cholestasis, gamma-glutamyltransferase increased, hepatic enzyme increased, liver function test abnormal, hepatocellular injury, liver injury, and transaminases increased. Detailed data were provided in Supplementary Table S4. The disproportionation analysis of the above adverse events suggested potential risks.

**FIGURE 3:**
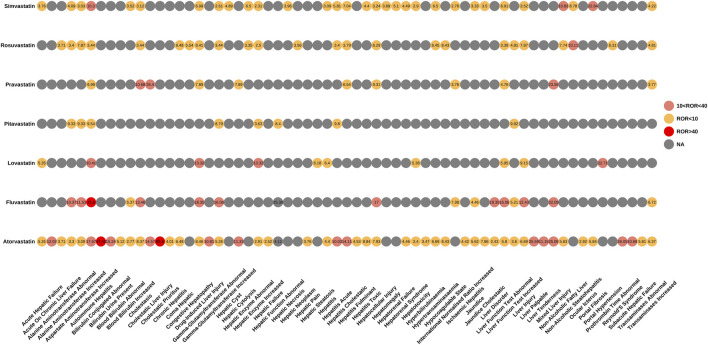
Signal heatmap of seven statins-related hepatic disorder adverse events calculated by ROR.

**FIGURE 4:**
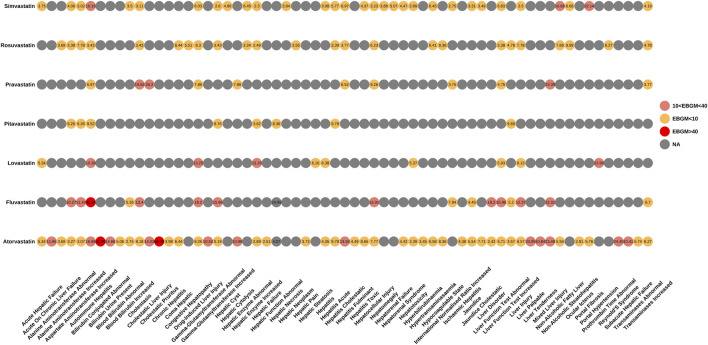
Signal heatmap of seven statins-related hepatic disorder adverse events calculated by EBGM.

We also performed an in-depth signal mining and analysis of preferred terms associated with various statin drugs using the ROR and EBGM methods. Figures 3, 4 presented the PT signal heat maps of seven statins obtained using the ROR and EBGM algorithms, respectively. Table 4 listed the top 5 PTs with the highest signal values for hepatic disorders caused by each class of statin drugs. In particular, atorvastatin showed significant signal values for cholestatic pruritus [ROR 85.3, 95% CI (32.16–226.21), EBGM 69.08, EBGM05 30.55] and bilirubbin conjugated abnormal [ROR 47.42, 95% CI (23.66–95.03), EBGM 41.99, EBGM05 23.47]. In addition, fluvastatin was notably associated with the risk of autoimmune hepatitis [ROR 41.6, 95% CI (22.99–75.25), EBGM 41.34, EBGM05 25.17]. The three adverse events mentioned above were the most prominent among the statins studied. In contrast, the signal value of hepatic enzyme increased [ROR 2.31, 95% CI (2–2.66), EBGM 2.3, EBGM05 2.04] caused by simvastatin was relatively low.

**TABLE 4:** The top five signal values of seven statins-related hepatic disorder adverse events at the PTs level.

	PT	Report number	ROR (95% Cl)	EBGM	EBGM05
Atorvastatin					
	Cholestatic Pruritus	5	85.3 (32.16–226.21)	69.08	30.55
	Bilirubin Conjugated Abnormal	9	47.42 (23.66–95.03)	41.99	23.47
	Reynold’S Syndrome	4	26.05 (9.44–71.89)	24.35	10.42
	Liver Palpable	9	25.59 (13.01–50.32)	23.95	13.60
	Mixed Liver Injury	133	25.08 (21.04–29.91)	23.49	20.28
Fluvastatin					
	Autoimmune Hepatitis	11	41.6 (22.99–75.25)	41.34	25.17
	Mixed Liver Injury	3	32.09 (10.33–99.66)	32.01	12.40
	Hepatic Function Abnormal	36	25.38 (18.26–35.27)	24.99	18.97
	Drug-Induced Liver Injury	19	18.35 (11.68–28.82)	18.20	12.47
	Hepatocellular Injury	12	17 (9.64–29.97)	16.91	10.52
Lovastatin					
	Portal Hypertension	3	22.71 (7.31–70.5)	22.66	8.78
	Drug-Induced Liver Injury	15	13.32 (8.02–22.14)	13.25	8.66
	Autoimmune Hepatitis	3	10.41 (3.35–32.31)	10.39	4.03
	Liver Injury	8	9.15 (4.57–18.33)	9.13	5.11
	Hepatitis	7	6.4 (3.05–13.43)	6.38	3.43
Pitavastatin					
	Liver Function Test Increased	12	9.92 (5.63–17.49)	9.89	6.15
	Hepatitis Acute	4	9.8 (3.67–26.13)	9.78	4.31
	Autoimmune Hepatitis	4	9.54 (3.58–25.43)	9.52	4.19
	Aspartate Aminotransferase Increased	32	9.33 (6.59–13.21)	9.26	6.92
	Gamma-Glutamyltransferase Increased	13	8.79 (5.1–15.16)	8.76	5.55
Pravastatin					
	Cholestatic Liver Injury	11	26.4 (14.59–47.79)	26.20	15.95
	Mixed Liver Injury	13	23.55 (13.65–40.64)	23.39	14.81
	Cholestasis	47	10.68 (8.01–14.22)	10.62	8.35
	Hepatocellular Injury	39	9.31 (6.79–12.75)	9.26	7.12
	Hepatic Cytolysis	21	7.89 (5.14–12.11)	7.86	5.49
Rosuvastatin					
	Non-Alcoholic Steatohepatitis	13	10.11 (5.85–17.46)	9.99	6.32
	Autoimmune Hepatitis	61	8.47 (6.58–10.9)	8.38	6.79
	Hypocoagulable State	9	8.43 (4.37–16.27)	8.36	4.82
	Drug-Induced Liver Injury	237	8.41 (7.39–9.56)	8.30	7.46
	Liver Injury	172	7.87 (6.77–9.15)	7.78	6.86
Simvastatin					
	Portal Fibrosis	6	22.84 (10.13–51.52)	22.14	11.21
	Non-Alcoholic Fatty Liver	8	10.83 (5.39–21.78)	10.68	5.95
	Autoimmune Hepatitis	85	10.3 (8.32–12.77)	10.16	8.49
	Non-Alcoholic Steatohepatitis	13	8.78 (5.08–15.18)	8.68	5.49
	Hepatitis Cholestatic	51	7.04 (5.34–9.27)	6.97	5.54

## Discussion

Statins are a cornerstone of lipid-lowering therapy and cardiovascular treatment. As their clinical applications continue to expand, researchers are gradually revealing the multiple efficiencies of this class of drugs. In addition to their core function of lowering cholesterol levels, statins have been shown to have antioxidant, endothelial-protective, and anti-inflammatory effects (Liu et al., 2019; Mollazadeh et al., 2021; Koushki et al., 2021) Furthermore, statins may also have the potential to reduce the risk of certain cancers (Jiang et al., 2021; Santoni et al., 2022) and improve the prognosis for patients with chronic kidney disease (Zhao et al., 2021). Recent research also suggests that statins may offer significant health benefits in patients with chronic liver disease and cirrhosis (Vargas et al., 2017).

The study of Montastruc JL demonstrated that statins may cause serious adverse reactions such as rhabdomyolysis, which warned us that the potential risks of statins should not be ignored (Montastruc, 2023). As the metabolic center of statins, the safety of the liver is particularly worth concerning. Current studies have pointed out that the use of statin drugs may lead to an abnormal increase in liver enzyme levels without symptoms, but these studies have not been deeply explored. When studying the impact of atorvastatin on the liver, Zhang H et al. found that the risk of hepatotoxicity may increase by 1.3–1.5 times after using the drug (Zhang et al., 2019). In addition, there have been reports of potential liver adverse reactions that may occur following the use of fluvastatin and simvastatin (Alanazi et al., 2021; Björnsson, 2017). Therefore, this study is of significant practical importance and necessity.

This study found that statins-induced hepatic disorders showed no significant difference between males and females, predominantly occurring in individuals aged 65–85 years, likely due to the clinical indications of statins for cardiovascular diseases, which are more prevalent in this age group. Additionally, it was observed that statins have a relatively high safety profile and do not lead to a high fatality rate, which could contribute to their widespread use. It was particularly important to note that the fatality rate due to hepatic disorder caused by simvastatin was the highest among the seven statins involved in this study, consistent with the findings of Björnsson ES, who pointed out that there was a certain correlation between simvastatin-induced hepatotoxicity and death (Björnsson, 2017). Mikiewicz M. et al. also found that simvastatin may lead to abnormal glycogen and lipid metabolism, changes in cell membrane permeability, liver fibrosis, hepatitis, decreased liver cell function, and an increased risk of apoptosis (Mikiewicz et al., 2019). Therefore, these findings serve as a warning that when using simvastatin, liver conditions of patients should be closely monitored to enhance clinical safety. It is worth noting that atorvastatin had the highest number of hepatic disorder cases, the highest number of annual reports, and the highest number of PTs of hepatic disorder events in this study. Simvastatin and rosuvastatin also showed relatively higher numbers which may be related to their significant lipid-lowering efficacy, leading to their extensive clinical use (Zhang et al., 2020).

Among the various hepatic disorders reported, autoimmune hepatitis and drug-induced liver injury have emerged as notable signals. For instance, autoimmune antibodies such as anti-nuclear antibody (ANA) and anti-smooth muscle antibody (ASMA) may appear following the use of rosuvastatin (Sánchez et al., 2018). The use of simvastatin has been associated with changes in hepatocytes resembling autoimmune hepatitis, accompanied by the emergence of antinuclear antibodies (Mikiewicz et al., 2019). There have also been reports of positive ANA and anti-ribonucleoprotein (RNP) antibodies following the use of atorvastatin (Tse et al., 2023). Additionally, it is particularly noteworthy that the signal value for autoimmune hepatitis induced by fluvastatin use is relatively high. This finding is consistent with the observation of fluvastatin induced autoimmune hepatitis in clinical practice by Nakayama and Murashima (2011). These studies not only suggest that statins should be used cautiously in patients with autoimmune diseases, but also provided a basis for future in-depth research on the relationship between statins and immune response. At the same time, it also suggests that clinicians should strengthen the monitoring of patients’ immune function after the use of statins, and actively search for possible immune-related answers.

The significant signal strength in cholestatic pruritus and bilirubin conjugation abnormal indicates a potential for severe hepatic reactions. In past medical research, statin drugs have been proven to be associated with cholestatic pruritus (Averbukh et al., 2022). Li WK et al. shed light on how statins affect the metabolic processes of bile acids. They found that atorvastatin, a widely used statin, significantly affects the expression of a key rate-limiting enzyme CYP7A1 in the liver (Li et al., 2017), which plays a crucial role in the synthesis of bile acids. By increasing the expression of CYP7A1, atorvastatin is able to promote bile acid synthesis, which may be responsible for cholestasis and related symptoms. A clinical review study further provided a detailed description of the pattern of liver damage caused by statins (Averbukh et al., 2022). Clinicians should pay close attention to the potential bile secretion disorders caused by statins. Before prescribing statins, physicians should perform a baseline liver function assessment to ensure the patient is free of liver disease and suitable for statin treatment. It is equally important to regularly monitor patients’ liver function during treatment to make informed and rational treatment decisions.

This study utilized the FAERS database to conduct a systematic analysis of the hepatotoxicity of statin drugs. On the one hand, we identified potential adverse events of hepatic disorder that may be caused by statin drugs. On the other hand, we conducted a thorough investigation of adverse events with specific signals. This study provided valuable insights into the potential hepatotoxicity of statins and added a new theoretical basis for evaluating the safety of these widely used drugs. Additionally, our study pointed the way for future pharmacovigilance analyses and clinical trials, aiding in further validating our findings and elucidating the mechanisms behind hepatotoxicity. However, we must also acknowledge that this study has certain limitations. Firstly, since the FAERS database relies on spontaneous reporting, there may be issues with underreporting and misreporting, which means that the information in the database may not entirely reflect the full picture of adverse events that occur after the actual use of statin drugs. Secondly, the causal relationship between the identified adverse events and statin drugs has not been definitively confirmed. Thirdly, this study did not adequately control for the disease background and concurrent drug situations of the subjects included in the study, which are confounding factors. Therefore, the accurate assessment of the hepatotoxicity of statins requires more prospective studies for in-depth analysis and clinical validation.

## Conclusion

This study highlights the potential hepatotoxicity issues associated with statin drugs, particularly noting the high incidence rates of atorvastatin, simvastatin, and rosuvastatin, with fluvastatin exhibiting a particularly prominent overall signal value for hepatotoxicity. In addition, adverse reactions such as autoimmune hepatitis and drug-induced liver injury that may occur during statin use also pose potential safety risks. Particularly, the specific adverse reactions of cholestatic pruritus and bilirubin conjugation abnormal caused by statins deserve further attention.

## Data Availability

The raw data supporting the conclusions of this article will be made available by the authors, without undue reservation.
