# Supplementary material for: Hepatotoxicity of statins: a real-world study based on the US Food and Drug Administration Adverse Event Reporting System database
Source: Front Pharmacol. 2025 Jan 7;15:1502791. doi: 10.3389/fphar.2024.1502791 (PMC11747658; doi:10.3389/fphar.2024.1502791)
Supplement: Supplementary file 1 [file Table1.docx]

**Supplementary Table S1. PTs for all drug-related hepatic disorders events reported in the FAERS database.**

| NO. |  | Code | PT | SMQ | Scope |
| --- | --- | --- | --- | --- | --- |
| 1 |  | 10074561 | Acquired Antithrombin Iii Deficiency | Drug related hepatic disorders - comprehensive search | Narrow |
| 2 |  | 10086006 | Acquired Factor V Deficiency | Drug related hepatic disorders - comprehensive search | Narrow |
| 3 |  | 10082745 | Acquired Factor Viii Deficiency | Drug related hepatic disorders - comprehensive search | Narrow |
| 4 |  | 10082746 | Acquired Factor Xi Deficiency | Drug related hepatic disorders - comprehensive search | Narrow |
| 5 |  | 10080860 | Acquired Hepatocerebral Degeneration | Drug related hepatic disorders - comprehensive search | Narrow |
| 6 |  | 10068370 | Acquired Protein S Deficiency | Drug related hepatic disorders - comprehensive search | Narrow |
| 7 |  | 10066263 | Acute Graft Versus Host Disease In Liver | Drug related hepatic disorders - comprehensive search | Narrow |
| 8 |  | 10000804 | Acute Hepatic Failure | Drug related hepatic disorders - comprehensive search | Narrow |
| 9 |  | 10077305 | Acute On Chronic Liver Failure | Drug related hepatic disorders - comprehensive search | Narrow |
| 10 |  | 10001547 | Alanine Aminotransferase Abnormal | Drug related hepatic disorders - comprehensive search | Narrow |
| 11 |  | 10001551 | Alanine Aminotransferase Increased | Drug related hepatic disorders - comprehensive search | Narrow |
| 12 |  | 10071198 | Allergic Hepatitis | Drug related hepatic disorders - comprehensive search | Narrow |
| 13 |  | 10080576 | Alloimmune Hepatitis | Drug related hepatic disorders - comprehensive search | Narrow |
| 14 |  | 10001942 | Ammonia Abnormal | Drug related hepatic disorders - comprehensive search | Narrow |
| 15 |  | 10001946 | Ammonia Increased | Drug related hepatic disorders - comprehensive search | Narrow |
| 16 |  | 10077670 | Anti Factor X Activity Abnormal | Drug related hepatic disorders - comprehensive search | Narrow |
| 17 |  | 10077674 | Anti Factor X Activity Decreased | Drug related hepatic disorders - comprehensive search | Narrow |
| 18 |  | 10077671 | Anti Factor X Activity Increased | Drug related hepatic disorders - comprehensive search | Narrow |
| 19 |  | 10049547 | Antithrombin Iii Decreased | Drug related hepatic disorders - comprehensive search | Narrow |
| 20 |  | 10003445 | Ascites | Drug related hepatic disorders - comprehensive search | Narrow |
| 21 |  | 10003477 | Aspartate Aminotransferase Abnormal | Drug related hepatic disorders - comprehensive search | Narrow |
| 22 |  | 10003481 | Aspartate Aminotransferase Increased | Drug related hepatic disorders - comprehensive search | Narrow |
| 23 |  | 10082832 | Ast/Alt Ratio Abnormal | Drug related hepatic disorders - comprehensive search | Narrow |
| 24 |  | 10003547 | Asterixis | Drug related hepatic disorders - comprehensive search | Narrow |
| 25 |  | 10003827 | Autoimmune Hepatitis | Drug related hepatic disorders - comprehensive search | Narrow |
| 26 |  | 10068547 | Bacterascites | Drug related hepatic disorders - comprehensive search | Narrow |
| 27 |  | 10004269 | Benign Hepatic Neoplasm | Drug related hepatic disorders - comprehensive search | Narrow |
| 28 |  | 10077922 | Benign Hepatobiliary Neoplasm | Drug related hepatic disorders - comprehensive search | Narrow |
| 29 |  | 10051344 | Bile Output Abnormal | Drug related hepatic disorders - comprehensive search | Narrow |
| 30 |  | 10051343 | Bile Output Decreased | Drug related hepatic disorders - comprehensive search | Narrow |
| 31 |  | 10074150 | Biliary Ascites | Drug related hepatic disorders - comprehensive search | Narrow |
| 32 |  | 10004659 | Biliary Cirrhosis | Drug related hepatic disorders - comprehensive search | Narrow |
| 33 |  | 10004664 | Biliary Fibrosis | Drug related hepatic disorders - comprehensive search | Narrow |
| 34 |  | 10067718 | Bilirubin Conjugated Abnormal | Drug related hepatic disorders - comprehensive search | Narrow |
| 35 |  | 10004685 | Bilirubin Conjugated Increased | Drug related hepatic disorders - comprehensive search | Narrow |
| 36 |  | 10061009 | Bilirubin Excretion Disorder | Drug related hepatic disorders - comprehensive search | Narrow |
| 37 |  | 10077356 | Bilirubin Urine Present | Drug related hepatic disorders - comprehensive search | Narrow |
| 38 |  | 10004792 | Biopsy Liver Abnormal | Drug related hepatic disorders - comprehensive search | Narrow |
| 39 |  | 10058477 | Blood Bilirubin Abnormal | Drug related hepatic disorders - comprehensive search | Narrow |
| 40 |  | 10005364 | Blood Bilirubin Increased | Drug related hepatic disorders - comprehensive search | Narrow |
| 41 |  | 10005370 | Blood Bilirubin Unconjugated Increased | Drug related hepatic disorders - comprehensive search | Narrow |
| 42 |  | 10005518 | Blood Fibrinogen Abnormal | Drug related hepatic disorders - comprehensive search | Narrow |
| 43 |  | 10005520 | Blood Fibrinogen Decreased | Drug related hepatic disorders - comprehensive search | Narrow |
| 44 |  | 10005818 | Blood Thrombin Abnormal | Drug related hepatic disorders - comprehensive search | Narrow |
| 45 |  | 10005820 | Blood Thrombin Decreased | Drug related hepatic disorders - comprehensive search | Narrow |
| 46 |  | 10005824 | Blood Thromboplastin Abnormal | Drug related hepatic disorders - comprehensive search | Narrow |
| 47 |  | 10005826 | Blood Thromboplastin Decreased | Drug related hepatic disorders - comprehensive search | Narrow |
| 48 |  | 10006408 | Bromosulphthalein Test Abnormal | Drug related hepatic disorders - comprehensive search | Narrow |
| 49 |  | 10082480 | Cardiohepatic Syndrome | Drug related hepatic disorders - comprehensive search | Narrow |
| 50 |  | 10077020 | Child-Pugh-Turcotte Score Abnormal | Drug related hepatic disorders - comprehensive search | Narrow |
| 51 |  | 10068287 | Child-Pugh-Turcotte Score Increased | Drug related hepatic disorders - comprehensive search | Narrow |
| 52 |  | 10048611 | Cholaemia | Drug related hepatic disorders - comprehensive search | Narrow |
| 53 |  | 10077861 | Cholangiosarcoma | Drug related hepatic disorders - comprehensive search | Narrow |
| 54 |  | 10008635 | Cholestasis | Drug related hepatic disorders - comprehensive search | Narrow |
| 55 |  | 10067969 | Cholestatic Liver Injury | Drug related hepatic disorders - comprehensive search | Narrow |
| 56 |  | 10064190 | Cholestatic Pruritus | Drug related hepatic disorders - comprehensive search | Narrow |
| 57 |  | 10072160 | Chronic Graft Versus Host Disease In Liver | Drug related hepatic disorders - comprehensive search | Narrow |
| 58 |  | 10057573 | Chronic Hepatic Failure | Drug related hepatic disorders - comprehensive search | Narrow |
| 59 |  | 10008909 | Chronic Hepatitis | Drug related hepatic disorders - comprehensive search | Narrow |
| 60 |  | 10009736 | Coagulation Factor Decreased | Drug related hepatic disorders - comprehensive search | Narrow |
| 61 |  | 10061770 | Coagulation Factor Ix Level Abnormal | Drug related hepatic disorders - comprehensive search | Narrow |
| 62 |  | 10009746 | Coagulation Factor Ix Level Decreased | Drug related hepatic disorders - comprehensive search | Narrow |
| 63 |  | 10061771 | Coagulation Factor V Level Abnormal | Drug related hepatic disorders - comprehensive search | Narrow |
| 64 |  | 10009754 | Coagulation Factor V Level Decreased | Drug related hepatic disorders - comprehensive search | Narrow |
| 65 |  | 10061772 | Coagulation Factor Vii Level Abnormal | Drug related hepatic disorders - comprehensive search | Narrow |
| 66 |  | 10009761 | Coagulation Factor Vii Level Decreased | Drug related hepatic disorders - comprehensive search | Narrow |
| 67 |  | 10061774 | Coagulation Factor X Level Abnormal | Drug related hepatic disorders - comprehensive search | Narrow |
| 68 |  | 10009775 | Coagulation Factor X Level Decreased | Drug related hepatic disorders - comprehensive search | Narrow |
| 69 |  | 10010075 | Coma Hepatic | Drug related hepatic disorders - comprehensive search | Narrow |
| 70 |  | 10076215 | Computerised Tomogram Liver | Drug related hepatic disorders - comprehensive search | Narrow |
| 71 |  | 10078360 | Computerised Tomogram Liver Abnormal | Drug related hepatic disorders - comprehensive search | Narrow |
| 72 |  | 10084058 | Congestive Hepatopathy | Drug related hepatic disorders - comprehensive search | Narrow |
| 73 |  | 10063075 | Cryptogenic Cirrhosis | Drug related hepatic disorders - comprehensive search | Narrow |
| 74 |  | 10071265 | Diabetic Hepatopathy | Drug related hepatic disorders - comprehensive search | Narrow |
| 75 |  | 10072268 | Drug-Induced Liver Injury | Drug related hepatic disorders - comprehensive search | Narrow |
| 76 |  | 10051010 | Duodenal Varices | Drug related hepatic disorders - comprehensive search | Narrow |
| 77 |  | 10052285 | Focal Nodular Hyperplasia | Drug related hepatic disorders - comprehensive search | Narrow |
| 78 |  | 10052554 | Foetor Hepaticus | Drug related hepatic disorders - comprehensive search | Narrow |
| 79 |  | 10072319 | Gallbladder Varices | Drug related hepatic disorders - comprehensive search | Narrow |
| 80 |  | 10017688 | Gamma-Glutamyltransferase Abnormal | Drug related hepatic disorders - comprehensive search | Narrow |
| 81 |  | 10017693 | Gamma-Glutamyltransferase Increased | Drug related hepatic disorders - comprehensive search | Narrow |
| 82 |  | 10051012 | Gastric Varices | Drug related hepatic disorders - comprehensive search | Narrow |
| 83 |  | 10057572 | Gastric Varices Haemorrhage | Drug related hepatic disorders - comprehensive search | Narrow |
| 84 |  | 10064676 | Graft Versus Host Disease In Liver | Drug related hepatic disorders - comprehensive search | Narrow |
| 85 |  | 10018821 | Haemangioma Of Liver | Drug related hepatic disorders - comprehensive search | Narrow |
| 86 |  | 10067796 | Haemorrhagic Hepatic Cyst | Drug related hepatic disorders - comprehensive search | Narrow |
| 87 |  | 10019621 | Hepaplastin Abnormal | Drug related hepatic disorders - comprehensive search | Narrow |
| 88 |  | 10019622 | Hepaplastin Decreased | Drug related hepatic disorders - comprehensive search | Narrow |
| 89 |  | 10061997 | Hepatectomy | Drug related hepatic disorders - comprehensive search | Narrow |
| 90 |  | 10019629 | Hepatic Adenoma | Drug related hepatic disorders - comprehensive search | Narrow |
| 91 |  | 10067388 | Hepatic Angiosarcoma | Drug related hepatic disorders - comprehensive search | Narrow |
| 92 |  | 10068997 | Hepatic Artery Flow Decreased | Drug related hepatic disorders - comprehensive search | Narrow |
| 93 |  | 10019637 | Hepatic Atrophy | Drug related hepatic disorders - comprehensive search | Narrow |
| 94 |  | 10065274 | Hepatic Calcification | Drug related hepatic disorders - comprehensive search | Narrow |
| 95 |  | 10073069 | Hepatic Neoplasm Malignant Resectable | Drug related hepatic disorders - comprehensive search | Narrow |
| 96 |  | 10055110 | Hepatic Cancer Metastatic | Drug related hepatic disorders - comprehensive search | Narrow |
| 97 |  | 10073070 | Hepatic Cancer Recurrent | Drug related hepatic disorders - comprehensive search | Narrow |
| 98 |  | 10059318 | Hepatic Cancer Stage I | Drug related hepatic disorders - comprehensive search | Narrow |
| 99 |  | 10059319 | Hepatic Cancer Stage Ii | Drug related hepatic disorders - comprehensive search | Narrow |
| 100 |  | 10059324 | Hepatic Cancer Stage Iii | Drug related hepatic disorders - comprehensive search | Narrow |
| 101 |  | 10059325 | Hepatic Cancer Stage Iv | Drug related hepatic disorders - comprehensive search | Narrow |
| 102 |  | 10019641 | Hepatic Cirrhosis | Drug related hepatic disorders - comprehensive search | Narrow |
| 103 |  | 10019646 | Hepatic Cyst | Drug related hepatic disorders - comprehensive search | Narrow |
| 104 |  | 10053973 | Hepatic Cyst Ruptured | Drug related hepatic disorders - comprehensive search | Narrow |
| 105 |  | 10049199 | Cytolytic Hepatitis | Drug related hepatic disorders - comprehensive search | Narrow |
| 106 |  | 10019660 | Minimal Hepatic Encephalopathy | Drug related hepatic disorders - comprehensive search | Narrow |
| 107 |  | 10066599 | Hepatic Encephalopathy Prophylaxis | Drug related hepatic disorders - comprehensive search | Narrow |
| 108 |  | 10062685 | Hepatic Enzyme Abnormal | Drug related hepatic disorders - comprehensive search | Narrow |
| 109 |  | 10060794 | Hepatic Enzyme Decreased | Drug related hepatic disorders - comprehensive search | Narrow |
| 110 |  | 10060795 | Hepatic Enzyme Increased | Drug related hepatic disorders - comprehensive search | Narrow |
| 111 |  | 10019663 | Hepatic Failure | Drug related hepatic disorders - comprehensive search | Narrow |
| 112 |  | 10019668 | Hepatic Fibrosis | Drug related hepatic disorders - comprehensive search | Narrow |
| 113 |  | 10019670 | Hepatic Function Abnormal | Drug related hepatic disorders - comprehensive search | Narrow |
| 114 |  | 10054885 | Hepatic Haemangioma Rupture | Drug related hepatic disorders - comprehensive search | Narrow |
| 115 |  | 10079685 | Hepatic Hamartoma | Drug related hepatic disorders - comprehensive search | Narrow |
| 116 |  | 10067365 | Hepatic Hydrothorax | Drug related hepatic disorders - comprehensive search | Narrow |
| 117 |  | 10076254 | Hepatic Hypertrophy | Drug related hepatic disorders - comprehensive search | Narrow |
| 118 |  | 10084751 | Hepatic Hypoperfusion | Drug related hepatic disorders - comprehensive search | Narrow |
| 119 |  | 10064668 | Hepatic Infiltration Eosinophilic | Drug related hepatic disorders - comprehensive search | Narrow |
| 120 |  | 10061998 | Hepatic Lesion | Drug related hepatic disorders - comprehensive search | Narrow |
| 121 |  | 10057110 | Hepatic Mass | Drug related hepatic disorders - comprehensive search | Narrow |
| 122 |  | 10019692 | Hepatic Necrosis | Drug related hepatic disorders - comprehensive search | Narrow |
| 123 |  | 10019695 | Hepatic Neoplasm | Drug related hepatic disorders - comprehensive search | Narrow |
| 124 |  | 10085864 | Hepatic Neuroendocrine Tumour | Drug related hepatic disorders - comprehensive search | Narrow |
| 125 |  | 10019705 | Hepatic Pain | Drug related hepatic disorders - comprehensive search | Narrow |
| 126 |  | 10066244 | Hepatic Sequestration | Drug related hepatic disorders - comprehensive search | Narrow |
| 127 |  | 10077215 | Hepatic Steato-Fibrosis | Drug related hepatic disorders - comprehensive search | Narrow |
| 128 |  | 10019708 | Hepatic Steatosis | Drug related hepatic disorders - comprehensive search | Narrow |
| 129 |  | 10068358 | Hepatic Vascular Resistance Increased | Drug related hepatic disorders - comprehensive search | Narrow |
| 130 |  | 10019717 | Hepatitis | Drug related hepatic disorders - comprehensive search | Narrow |
| 131 |  | 10019727 | Hepatitis Acute | Drug related hepatic disorders - comprehensive search | Narrow |
| 132 |  | 10019754 | Hepatitis Cholestatic | Drug related hepatic disorders - comprehensive search | Narrow |
| 133 |  | 10019755 | Hepatitis Chronic Active | Drug related hepatic disorders - comprehensive search | Narrow |
| 134 |  | 10019759 | Hepatitis Chronic Persistent | Drug related hepatic disorders - comprehensive search | Narrow |
| 135 |  | 10019772 | Hepatitis Fulminant | Drug related hepatic disorders - comprehensive search | Narrow |
| 136 |  | 10019795 | Hepatitis Toxic | Drug related hepatic disorders - comprehensive search | Narrow |
| 137 |  | 10073073 | Malignant Hepatobiliary Neoplasm | Drug related hepatic disorders - comprehensive search | Narrow |
| 138 |  | 10073074 | Hepatobiliary Carcinoma In Situ | Drug related hepatic disorders - comprehensive search | Narrow |
| 139 |  | 10079889 | Hepatobiliary Cyst | Drug related hepatic disorders - comprehensive search | Narrow |
| 140 |  | 10062000 | Hepatobiliary Disease | Drug related hepatic disorders - comprehensive search | Narrow |
| 141 |  | 10061203 | Hepatobiliary Neoplasm | Drug related hepatic disorders - comprehensive search | Narrow |
| 142 |  | 10066195 | Hepatobiliary Scan Abnormal | Drug related hepatic disorders - comprehensive search | Narrow |
| 143 |  | 10062001 | Hepatoblastoma | Drug related hepatic disorders - comprehensive search | Narrow |
| 144 |  | 10019823 | Hepatoblastoma Recurrent | Drug related hepatic disorders - comprehensive search | Narrow |
| 145 |  | 10073071 | Hepatocellular Carcinoma | Drug related hepatic disorders - comprehensive search | Narrow |
| 146 |  | 10053244 | Hepatocellular Foamy Cell Syndrome | Drug related hepatic disorders - comprehensive search | Narrow |
| 147 |  | 10019837 | Hepatocellular Injury | Drug related hepatic disorders - comprehensive search | Narrow |
| 148 |  | 10019842 | Hepatomegaly | Drug related hepatic disorders - comprehensive search | Narrow |
| 149 |  | 10052274 | Hepatopulmonary Syndrome | Drug related hepatic disorders - comprehensive search | Narrow |
| 150 |  | 10019845 | Hepatorenal Failure | Drug related hepatic disorders - comprehensive search | Narrow |
| 151 |  | 10019846 | Hepatorenal Syndrome | Drug related hepatic disorders - comprehensive search | Narrow |
| 152 |  | 10019847 | Hepatosplenomegaly | Drug related hepatic disorders - comprehensive search | Narrow |
| 153 |  | 10019851 | Hepatotoxicity | Drug related hepatic disorders - comprehensive search | Narrow |
| 154 |  | 10020575 | Hyperammonaemia | Drug related hepatic disorders - comprehensive search | Narrow |
| 155 |  | 10020578 | Hyperbilirubinaemia | Drug related hepatic disorders - comprehensive search | Narrow |
| 156 |  | 10051924 | Hypercholia | Drug related hepatic disorders - comprehensive search | Narrow |
| 157 |  | 10074737 | Hyperfibrinolysis | Drug related hepatic disorders - comprehensive search | Narrow |
| 158 |  | 10068237 | Hypertransaminasaemia | Drug related hepatic disorders - comprehensive search | Narrow |
| 159 |  | 10020973 | Hypocoagulable State | Drug related hepatic disorders - comprehensive search | Narrow |
| 160 |  | 10051125 | Hypofibrinogenaemia | Drug related hepatic disorders - comprehensive search | Narrow |
| 161 |  | 10021085 | Hypoprothrombinaemia | Drug related hepatic disorders - comprehensive search | Narrow |
| 162 |  | 10058517 | Hypothrombinaemia | Drug related hepatic disorders - comprehensive search | Narrow |
| 163 |  | 10021209 | Icterus Index Increased | Drug related hepatic disorders - comprehensive search | Narrow |
| 164 |  | 10083406 | Immune-Mediated Cholangitis | Drug related hepatic disorders - comprehensive search | Narrow |
| 165 |  | 10083521 | Immune-Mediated Hepatic Disorder | Drug related hepatic disorders - comprehensive search | Narrow |
| 166 |  | 10078962 | Immune-Mediated Hepatitis | Drug related hepatic disorders - comprehensive search | Narrow |
| 167 |  | 10022592 | International Normalised Ratio Abnormal | Drug related hepatic disorders - comprehensive search | Narrow |
| 168 |  | 10022595 | International Normalised Ratio Increased | Drug related hepatic disorders - comprehensive search | Narrow |
| 169 |  | 10071502 | Intestinal Varices | Drug related hepatic disorders - comprehensive search | Narrow |
| 170 |  | 10078058 | Intestinal Varices Haemorrhage | Drug related hepatic disorders - comprehensive search | Narrow |
| 171 |  | 10023025 | Ischaemic Hepatitis | Drug related hepatic disorders - comprehensive search | Narrow |
| 172 |  | 10023126 | Jaundice | Drug related hepatic disorders - comprehensive search | Narrow |
| 173 |  | 10023129 | Jaundice Cholestatic | Drug related hepatic disorders - comprehensive search | Narrow |
| 174 |  | 10023136 | Jaundice Hepatocellular | Drug related hepatic disorders - comprehensive search | Narrow |
| 175 |  | 10023321 | Kayser-Fleischer Ring | Drug related hepatic disorders - comprehensive search | Narrow |
| 176 |  | 10050842 | Liver Carcinoma Ruptured | Drug related hepatic disorders - comprehensive search | Narrow |
| 177 |  | 10076640 | Liver Dialysis | Drug related hepatic disorders - comprehensive search | Narrow |
| 178 |  | 10024670 | Liver Disorder | Drug related hepatic disorders - comprehensive search | Narrow |
| 179 |  | 10024690 | Liver Function Test Abnormal | Drug related hepatic disorders - comprehensive search | Narrow |
| 180 |  | 10077677 | Liver Function Test Decreased | Drug related hepatic disorders - comprehensive search | Narrow |
| 181 |  | 10077692 | Liver Function Test Increased | Drug related hepatic disorders - comprehensive search | Narrow |
| 182 |  | 10052550 | Liver Induration | Drug related hepatic disorders - comprehensive search | Narrow |
| 183 |  | 10067125 | Liver Injury | Drug related hepatic disorders - comprehensive search | Narrow |
| 184 |  | 10062040 | Liver Operation | Drug related hepatic disorders - comprehensive search | Narrow |
| 185 |  | 10075895 | Liver Palpable | Drug related hepatic disorders - comprehensive search | Narrow |
| 186 |  | 10061947 | Liver Scan Abnormal | Drug related hepatic disorders - comprehensive search | Narrow |
| 187 |  | 10024712 | Liver Tenderness | Drug related hepatic disorders - comprehensive search | Narrow |
| 188 |  | 10024714 | Liver Transplant | Drug related hepatic disorders - comprehensive search | Narrow |
| 189 |  | 10025129 | Lupoid Hepatic Cirrhosis | Drug related hepatic disorders - comprehensive search | Narrow |
| 190 |  | 10067737 | Lupus Hepatitis | Drug related hepatic disorders - comprehensive search | Narrow |
| 191 |  | 10085121 | Magnetic Resonance Imaging Hepatobiliary Abnormal | Drug related hepatic disorders - comprehensive search | Narrow |
| 192 |  | 10064712 | Mitochondrial Aspartate Aminotransferase Increased | Drug related hepatic disorders - comprehensive search | Narrow |
| 193 |  | 10027761 | Mixed Hepatocellular Cholangiocarcinoma | Drug related hepatic disorders - comprehensive search | Narrow |
| 194 |  | 10066758 | Mixed Hepatocellular-Cholestatic Injury | Drug related hepatic disorders - comprehensive search | Narrow |
| 195 |  | 10066869 | Molar Ratio Of Total Branched-Chain Amino Acid To Tyrosine | Drug related hepatic disorders - comprehensive search | Narrow |
| 196 |  | 10051081 | Nodular Regenerative Hyperplasia | Drug related hepatic disorders - comprehensive search | Narrow |
| 197 |  | 10029530 | Nonalcoholic Fatty Liver Disease | Drug related hepatic disorders - comprehensive search | Narrow |
| 198 |  | 10053219 | Non-Alcoholic Steatohepatitis | Drug related hepatic disorders - comprehensive search | Narrow |
| 199 |  | 10077259 | Non-Cirrhotic Portal Hypertension | Drug related hepatic disorders - comprehensive search | Narrow |
| 200 |  | 10058117 | Ocular Icterus | Drug related hepatic disorders - comprehensive search | Narrow |
| 201 |  | 10049631 | Oedema Due To Hepatic Disease | Drug related hepatic disorders - comprehensive search | Narrow |
| 202 |  | 10030210 | Oesophageal Varices Haemorrhage | Drug related hepatic disorders - comprehensive search | Narrow |
| 203 |  | 10074151 | Parenteral Nutrition Associated Liver Disease | Drug related hepatic disorders - comprehensive search | Narrow |
| 204 |  | 10054125 | Perihepatic Discomfort | Drug related hepatic disorders - comprehensive search | Narrow |
| 205 |  | 10073215 | Peripancreatic Varices | Drug related hepatic disorders - comprehensive search | Narrow |
| 206 |  | 10074726 | Portal Fibrosis | Drug related hepatic disorders - comprehensive search | Narrow |
| 207 |  | 10036200 | Portal Hypertension | Drug related hepatic disorders - comprehensive search | Narrow |
| 208 |  | 10079446 | Portal Hypertensive Colopathy | Drug related hepatic disorders - comprehensive search | Narrow |
| 209 |  | 10068923 | Portal Hypertensive Enteropathy | Drug related hepatic disorders - comprehensive search | Narrow |
| 210 |  | 10050897 | Portal Hypertensive Gastropathy | Drug related hepatic disorders - comprehensive search | Narrow |
| 211 |  | 10073979 | Portal Vein Cavernous Transformation | Drug related hepatic disorders - comprehensive search | Narrow |
| 212 |  | 10073209 | Portal Vein Dilatation | Drug related hepatic disorders - comprehensive search | Narrow |
| 213 |  | 10067281 | Portopulmonary Hypertension | Drug related hepatic disorders - comprehensive search | Narrow |
| 214 |  | 10080429 | Primary Biliary Cholangitis | Drug related hepatic disorders - comprehensive search | Narrow |
| 215 |  | 10037005 | Protein C Decreased | Drug related hepatic disorders - comprehensive search | Narrow |
| 216 |  | 10051736 | Protein S Abnormal | Drug related hepatic disorders - comprehensive search | Narrow |
| 217 |  | 10051120 | Protein S Decreased | Drug related hepatic disorders - comprehensive search | Narrow |
| 218 |  | 10037048 | Prothrombin Level Abnormal | Drug related hepatic disorders - comprehensive search | Narrow |
| 219 |  | 10037050 | Prothrombin Level Decreased | Drug related hepatic disorders - comprehensive search | Narrow |
| 220 |  | 10037057 | Prothrombin Time Abnormal | Drug related hepatic disorders - comprehensive search | Narrow |
| 221 |  | 10037063 | Prothrombin Time Prolonged | Drug related hepatic disorders - comprehensive search | Narrow |
| 222 |  | 10061918 | Prothrombin Time Ratio Abnormal | Drug related hepatic disorders - comprehensive search | Narrow |
| 223 |  | 10037068 | Prothrombin Time Ratio Increased | Drug related hepatic disorders - comprehensive search | Narrow |
| 224 |  | 10051015 | Radiation Hepatitis | Drug related hepatic disorders - comprehensive search | Narrow |
| 225 |  | 10052279 | Renal And Liver Transplant | Drug related hepatic disorders - comprehensive search | Narrow |
| 226 |  | 10067338 | Retrograde Portal Vein Flow | Drug related hepatic disorders - comprehensive search | Narrow |
| 227 |  | 10039012 | Reye'S Syndrome | Drug related hepatic disorders - comprehensive search | Narrow |
| 228 |  | 10070953 | Reynold'S Syndrome | Drug related hepatic disorders - comprehensive search | Narrow |
| 229 |  | 10067823 | Splenic Varices | Drug related hepatic disorders - comprehensive search | Narrow |
| 230 |  | 10068662 | Splenic Varices Haemorrhage | Drug related hepatic disorders - comprehensive search | Narrow |
| 231 |  | 10061135 | Spontaneous Bacterial Peritonitis | Drug related hepatic disorders - comprehensive search | Narrow |
| 232 |  | 10076331 | Steatohepatitis | Drug related hepatic disorders - comprehensive search | Narrow |
| 233 |  | 10056956 | Subacute Hepatic Failure | Drug related hepatic disorders - comprehensive search | Narrow |
| 234 |  | 10051319 | Thrombin Time Abnormal | Drug related hepatic disorders - comprehensive search | Narrow |
| 235 |  | 10051390 | Thrombin Time Prolonged | Drug related hepatic disorders - comprehensive search | Narrow |
| 236 |  | 10064558 | Total Bile Acids Increased | Drug related hepatic disorders - comprehensive search | Narrow |
| 237 |  | 10062688 | Transaminases Abnormal | Drug related hepatic disorders - comprehensive search | Narrow |
| 238 |  | 10054889 | Transaminases Increased | Drug related hepatic disorders - comprehensive search | Narrow |
| 239 |  | 10045428 | Ultrasound Liver Abnormal | Drug related hepatic disorders - comprehensive search | Narrow |
| 240 |  | 10050792 | Urine Bilirubin Increased | Drug related hepatic disorders - comprehensive search | Narrow |
| 241 |  | 10056091 | Varices Oesophageal | Drug related hepatic disorders - comprehensive search | Narrow |
| 242 |  | 10072284 | Varicose Veins Of Abdominal Wall | Drug related hepatic disorders - comprehensive search | Narrow |
| 243 |  | 10078438 | White Nipple Sign | Drug related hepatic disorders - comprehensive search | Narrow |
| 244 |  | 10056536 | X-Ray Hepatobiliary Abnormal | Drug related hepatic disorders - comprehensive search | Narrow |

**Supplementary Table S2. Algorithm for disproportionate analyses.**

| Drugs | Hepatic disorder adverse event cases | Non hepatic disorder adverse events cases |
| --- | --- | --- |
| Target drug | a | b |
| All other drugs | c | d |

**Supplementary Table S3. Calculation of reporting odds ratio (ROR) and Empirical Bayes Geometric Mean (EBGM) and their threshold**

| Method | Formula | Threshold |
| --- | --- | --- |
| ROR | ROR=$\frac{(a/c)}{(b/d)}=\frac{ad}{bc}$ | a≥3 and 95%CI(lower limit)>1 |
|  | SE(lnROR)=$\sqrt{(\frac{1}{a}+\frac{1}{b}+\frac{1}{c}+\frac{1}{d})}$ |  |
|  | 95%CI=℮^ln(ROR)±1.96^ |  |
| EBGM | EBGM= | EBGM05>2 |
|  | 95%CI=℮^ln（EBGM)±1.96^ |  |

**Supplementary Table S4.** **The signal values of seven statins-related hepatic disorder adverse events at the PTs level.**

|  | PT | Report number | ROR(95%Cl) | EBGM | EBGM05 |
| --- | --- | --- | --- | --- | --- |
| Atorvastatin |  |  |  |  |  |
|  | Drug-Induced Liver Injury | 521 | 8.46 ( 7.75 - 9.23 ) | 8.26 | 7.68 |
|  | Alanine Aminotransferase Increased | 500 | 3.3 ( 3.02 - 3.6 ) | 3.27 | 3.04 |
|  | Hepatic Enzyme Increased | 458 | 2.91 ( 2.65 - 3.19 ) | 2.89 | 2.67 |
|  | Liver Function Test Abnormal | 429 | 5.8 ( 5.27 - 6.39 ) | 5.71 | 5.27 |
|  | Aspartate Aminotransferase Increased | 406 | 3.09 ( 2.8 - 3.41 ) | 3.07 | 2.83 |
|  | Jaundice | 376 | 5.62 ( 5.08 - 6.23 ) | 5.54 | 5.09 |
|  | Cholestasis | 369 | 8.37 ( 7.55 - 9.28 ) | 8.18 | 7.51 |
|  | Hepatic Function Abnormal | 354 | 4.11 ( 3.7 - 4.57 ) | 4.07 | 3.73 |
|  | Transaminases Increased | 339 | 6.37 ( 5.72 - 7.1 ) | 6.27 | 5.73 |
|  | Hepatocellular Injury | 333 | 7.93 ( 7.11 - 8.84 ) | 7.77 | 7.09 |
|  | Liver Injury | 321 | 6.69 ( 5.99 - 7.47 ) | 6.57 | 5.99 |
|  | Hepatic Cytolysis | 299 | 11.33 ( 10.1 - 12.71 ) | 10.99 | 9.98 |
|  | Gamma-Glutamyltransferase Increased | 295 | 5.26 ( 4.69 - 5.91 ) | 5.19 | 4.72 |
|  | Autoimmune Hepatitis | 271 | 17.67 ( 15.64 - 19.96 ) | 16.86 | 15.22 |
|  | Hepatitis | 266 | 4.4 ( 3.9 - 4.97 ) | 4.35 | 3.93 |
|  | Liver Disorder | 256 | 2.43 ( 2.15 - 2.75 ) | 2.42 | 2.18 |
|  | Hepatitis Cholestatic | 191 | 14.11 ( 12.21 - 16.3 ) | 13.59 | 12.04 |
|  | Blood Bilirubin Increased | 186 | 2.77 ( 2.39 - 3.19 ) | 2.75 | 2.44 |
|  | Hepatic Failure | 186 | 2.52 ( 2.18 - 2.91 ) | 2.51 | 2.22 |
|  | Liver Function Test Increased | 166 | 3.6 ( 3.09 - 4.2 ) | 3.57 | 3.14 |
|  | Hepatitis Acute | 153 | 10.03 ( 8.54 - 11.78 ) | 9.78 | 8.55 |
|  | Mixed Liver Injury | 133 | 25.08 ( 21.04 - 29.91 ) | 23.49 | 20.28 |
|  | Hepatotoxicity | 124 | 2.4 ( 2.01 - 2.86 ) | 2.39 | 2.06 |
|  | Hypertransaminasaemia | 84 | 6.69 ( 5.39 - 8.3 ) | 6.58 | 5.49 |
|  | Hyperbilirubinaemia | 84 | 3.47 ( 2.8 - 4.3 ) | 3.45 | 2.88 |
|  | Jaundice Cholestatic | 68 | 7.86 ( 6.18 - 10 ) | 7.71 | 6.31 |
|  | Cholestatic Liver Injury | 60 | 14.57 ( 11.25 - 18.86 ) | 14.03 | 11.31 |
|  | Hepatitis Toxic | 57 | 8.84 ( 6.8 - 11.5 ) | 8.65 | 6.94 |
|  | Hepatic Pain | 39 | 3.76 ( 2.74 - 5.15 ) | 3.73 | 2.86 |
|  | Hepatitis Fulminant | 33 | 4.53 ( 3.22 - 6.39 ) | 4.49 | 3.37 |
|  | Ocular Icterus | 31 | 2.92 ( 2.05 - 4.16 ) | 2.91 | 2.16 |
|  | Hepatorenal Syndrome | 19 | 4.46 ( 2.84 - 7.01 ) | 4.42 | 3.03 |
|  | Blood Bilirubin Abnormal | 15 | 5.12 ( 3.08 - 8.53 ) | 5.06 | 3.31 |
|  | Gamma-Glutamyltransferase Abnormal | 14 | 10.81 ( 6.35 - 18.4 ) | 10.52 | 6.74 |
|  | Ischaemic Hepatitis | 14 | 4.42 ( 2.61 - 7.49 ) | 4.38 | 2.82 |
|  | Bilirubin Conjugated Abnormal | 9 | 47.42 ( 23.66 - 95.03 ) | 41.99 | 23.47 |
|  | Liver Palpable | 9 | 25.59 ( 13.01 - 50.32 ) | 23.95 | 13.60 |
|  | Liver Tenderness | 8 | 11.15 ( 5.52 - 22.54 ) | 10.84 | 6.02 |
|  | Transaminases Abnormal | 8 | 5.81 ( 2.89 - 11.69 ) | 5.74 | 3.20 |
|  | Non-Alcoholic Fatty Liver | 8 | 5.63 ( 2.8 - 11.32 ) | 5.56 | 3.10 |
|  | Chronic Hepatitis | 8 | 4.01 ( 2 - 8.06 ) | 3.98 | 2.22 |
|  | Cholestatic Pruritus | 5 | 85.3 ( 32.16 - 226.21 ) | 69.08 | 30.55 |
|  | Acute On Chronic Liver Failure | 5 | 12.02 ( 4.93 - 29.31 ) | 11.66 | 5.53 |
|  | Reynold'S Syndrome | 4 | 26.05 ( 9.44 - 71.89 ) | 24.35 | 10.42 |
|  | Bilirubin Urine Present | 4 | 15.24 ( 5.6 - 41.47 ) | 14.66 | 6.35 |
|  | Subacute Hepatic Failure | 4 | 10.69 ( 3.96 - 28.91 ) | 10.41 | 4.53 |
|  | Portal Fibrosis | 3 | 5.84 ( 1.87 - 18.28 ) | 5.76 | 2.22 |
| Fluvastatin |  |  |  |  |  |
|  | Hepatic Function Abnormal | 36 | 25.38 ( 18.26 - 35.27 ) | 24.99 | 18.97 |
|  | Alanine Aminotransferase Increased | 26 | 10.37 ( 7.05 - 15.26 ) | 10.27 | 7.43 |
|  | Aspartate Aminotransferase Increased | 25 | 11.53 ( 7.77 - 17.1 ) | 11.42 | 8.21 |
|  | Drug-Induced Liver Injury | 19 | 18.35 ( 11.68 - 28.82 ) | 18.20 | 12.47 |
|  | Liver Disorder | 18 | 10.36 ( 6.52 - 16.47 ) | 10.29 | 6.98 |
|  | Gamma-Glutamyltransferase Increased | 15 | 16.06 ( 9.66 - 26.68 ) | 15.96 | 10.43 |
|  | Liver Function Test Abnormal | 15 | 12.14 ( 7.31 - 20.18 ) | 12.07 | 7.89 |
|  | Hepatocellular Injury | 12 | 17 ( 9.64 - 29.97 ) | 16.91 | 10.52 |
|  | Autoimmune Hepatitis | 11 | 41.6 ( 22.99 - 75.25 ) | 41.34 | 25.17 |
|  | Cholestasis | 10 | 13.46 ( 7.23 - 25.05 ) | 13.40 | 7.97 |
|  | Liver Injury | 10 | 12.43 ( 6.68 - 23.13 ) | 12.37 | 7.36 |
|  | International Normalised Ratio Increased | 10 | 7.98 ( 4.29 - 14.84 ) | 7.94 | 4.72 |
|  | Transaminases Increased | 6 | 6.72 ( 3.02 - 14.97 ) | 6.70 | 3.43 |
|  | Blood Bilirubin Increased | 6 | 5.37 ( 2.41 - 11.97 ) | 5.36 | 2.74 |
|  | Jaundice | 5 | 4.46 ( 1.85 - 10.72 ) | 4.45 | 2.14 |
|  | Liver Function Test Increased | 4 | 5.21 ( 1.95 - 13.9 ) | 5.20 | 2.29 |
|  | Mixed Liver Injury | 3 | 32.09 ( 10.33 - 99.66 ) | 32.01 | 12.40 |
| Lovastatin |  |  |  |  |  |
|  | Drug-Induced Liver Injury | 15 | 13.32 ( 8.02 - 22.14 ) | 13.25 | 8.66 |
|  | Hepatic Enzyme Increased | 13 | 4.58 ( 2.66 - 7.9 ) | 4.56 | 2.89 |
|  | Liver Injury | 8 | 9.15 ( 4.57 - 18.33 ) | 9.13 | 5.11 |
|  | Liver Function Test Abnormal | 8 | 5.95 ( 2.97 - 11.91 ) | 5.93 | 3.32 |
|  | Hepatitis | 7 | 6.4 ( 3.05 - 13.43 ) | 6.38 | 3.43 |
|  | Hepatic Steatosis | 5 | 6.18 ( 2.57 - 14.85 ) | 6.16 | 2.96 |
|  | Hepatotoxicity | 5 | 5.38 ( 2.24 - 12.93 ) | 5.37 | 2.58 |
|  | Portal Hypertension | 3 | 22.71 ( 7.31 - 70.5 ) | 22.66 | 8.78 |
|  | Autoimmune Hepatitis | 3 | 10.41 ( 3.35 - 32.31 ) | 10.39 | 4.03 |
|  | Acute Hepatic Failure | 3 | 5.35 ( 1.72 - 16.6 ) | 5.34 | 2.07 |
| Pitavastatin |  |  |  |  |  |
|  | Aspartate Aminotransferase Increased | 32 | 9.33 ( 6.59 - 13.21 ) | 9.26 | 6.92 |
|  | Alanine Aminotransferase Increased | 25 | 6.29 ( 4.24 - 9.32 ) | 6.25 | 4.50 |
|  | Hepatic Function Abnormal | 19 | 8.4 ( 5.35 - 13.18 ) | 8.36 | 5.73 |
|  | Hepatic Enzyme Increased | 15 | 3.63 ( 2.18 - 6.02 ) | 3.62 | 2.37 |
|  | Gamma-Glutamyltransferase Increased | 13 | 8.79 ( 5.1 - 15.16 ) | 8.76 | 5.55 |
|  | Liver Function Test Increased | 12 | 9.92 ( 5.63 - 17.49 ) | 9.89 | 6.15 |
|  | Hepatitis Acute | 4 | 9.8 ( 3.67 - 26.13 ) | 9.78 | 4.31 |
|  | Autoimmune Hepatitis | 4 | 9.54 ( 3.58 - 25.43 ) | 9.52 | 4.19 |
| Pravastatin |  |  |  |  |  |
|  | Cholestasis | 47 | 10.68 ( 8.01 - 14.22 ) | 10.62 | 8.35 |
|  | Hepatocellular Injury | 39 | 9.31 ( 6.79 - 12.75 ) | 9.26 | 7.12 |
|  | Liver Function Test Abnormal | 35 | 4.76 ( 3.42 - 6.64 ) | 4.75 | 3.60 |
|  | International Normalised Ratio Increased | 28 | 3.76 ( 2.59 - 5.45 ) | 3.75 | 2.75 |
|  | Hepatic Cytolysis | 21 | 7.89 ( 5.14 - 12.11 ) | 7.86 | 5.49 |
|  | Transaminases Increased | 20 | 3.77 ( 2.43 - 5.85 ) | 3.77 | 2.61 |
|  | Drug-Induced Liver Injury | 20 | 3.23 ( 2.09 - 5.02 ) | 3.23 | 2.24 |
|  | Mixed Liver Injury | 13 | 23.55 ( 13.65 - 40.64 ) | 23.39 | 14.81 |
|  | Cholestatic Liver Injury | 11 | 26.4 ( 14.59 - 47.79 ) | 26.20 | 15.95 |
|  | Autoimmune Hepatitis | 11 | 6.99 ( 3.87 - 12.62 ) | 6.97 | 4.25 |
|  | Hepatitis Cholestatic | 9 | 6.54 ( 3.4 - 12.58 ) | 6.52 | 3.77 |
| Rosuvastatin |  |  |  |  |  |
|  | Drug-Induced Liver Injury | 237 | 8.41 ( 7.39 - 9.56 ) | 8.30 | 7.46 |
|  | Alanine Aminotransferase Increased | 234 | 3.4 ( 2.99 - 3.86 ) | 3.38 | 3.03 |
|  | Hepatic Enzyme Increased | 179 | 2.5 ( 2.16 - 2.9 ) | 2.49 | 2.20 |
|  | Liver Injury | 172 | 7.87 ( 6.77 - 9.15 ) | 7.78 | 6.86 |
|  | Aspartate Aminotransferase Increased | 159 | 2.66 ( 2.28 - 3.11 ) | 2.66 | 2.33 |
|  | Hepatic Function Abnormal | 133 | 3.39 ( 2.86 - 4.02 ) | 3.38 | 2.93 |
|  | Hepatocellular Injury | 121 | 6.29 ( 5.25 - 7.52 ) | 6.23 | 5.37 |
|  | Transaminases Increased | 117 | 4.81 ( 4.01 - 5.77 ) | 4.78 | 4.11 |
|  | Liver Function Test Increased | 72 | 3.44 ( 2.73 - 4.33 ) | 3.43 | 2.82 |
|  | Cholestasis | 70 | 3.44 ( 2.72 - 4.35 ) | 3.43 | 2.82 |
|  | Gamma-Glutamyltransferase Increased | 65 | 2.54 ( 1.99 - 3.23 ) | 2.53 | 2.06 |
|  | Autoimmune Hepatitis | 61 | 8.47 ( 6.58 - 10.9 ) | 8.38 | 6.79 |
|  | Hypertransaminasaemia | 37 | 6.45 ( 4.67 - 8.92 ) | 6.41 | 4.89 |
|  | Hepatitis Cholestatic | 24 | 3.79 ( 2.54 - 5.66 ) | 3.77 | 2.70 |
|  | Hepatitis Acute | 24 | 3.4 ( 2.28 - 5.08 ) | 3.39 | 2.42 |
|  | Hepatic Enzyme Abnormal | 18 | 3.35 ( 2.11 - 5.32 ) | 3.34 | 2.27 |
|  | Non-Alcoholic Steatohepatitis | 13 | 10.11 ( 5.85 - 17.46 ) | 9.99 | 6.32 |
|  | Hepatic Neoplasm | 13 | 3.56 ( 2.06 - 6.14 ) | 3.55 | 2.25 |
|  | Hypocoagulable State | 9 | 8.43 ( 4.37 - 16.27 ) | 8.36 | 4.82 |
|  | Congestive Hepatopathy | 9 | 5.54 ( 2.88 - 10.67 ) | 5.51 | 3.18 |
|  | Alanine Aminotransferase Abnormal | 8 | 3.71 ( 1.85 - 7.42 ) | 3.69 | 2.07 |
|  | Coma Hepatic | 6 | 6.48 ( 2.9 - 14.47 ) | 6.44 | 3.29 |
|  | Non-Alcoholic Fatty Liver | 5 | 7.74 ( 3.21 - 18.68 ) | 7.68 | 3.67 |
|  | Prothrombin Time Abnormal | 5 | 6.31 ( 2.62 - 15.22 ) | 6.27 | 3.00 |
| Simvastatin |  |  |  |  |  |
|  | Alanine Aminotransferase Increased | 323 | 4.09 ( 3.66 - 4.56 ) | 4.06 | 3.70 |
|  | Liver Function Test Abnormal | 267 | 6.91 ( 6.12 - 7.8 ) | 6.83 | 6.17 |
|  | Aspartate Aminotransferase Increased | 208 | 3.03 ( 2.65 - 3.48 ) | 3.02 | 2.69 |
|  | Drug-Induced Liver Injury | 198 | 6.09 ( 5.29 - 7 ) | 6.03 | 5.36 |
|  | Hepatic Enzyme Increased | 190 | 2.31 ( 2 - 2.66 ) | 2.30 | 2.04 |
|  | Transaminases Increased | 118 | 4.22 ( 3.52 - 5.05 ) | 4.19 | 3.60 |
|  | Jaundice | 117 | 3.33 ( 2.77 - 3.99 ) | 3.31 | 2.84 |
|  | International Normalised Ratio Increased | 109 | 2.76 ( 2.29 - 3.34 ) | 2.75 | 2.35 |
|  | Hepatitis | 98 | 3.09 ( 2.54 - 3.77 ) | 3.08 | 2.61 |
|  | Liver Injury | 89 | 3.52 ( 2.86 - 4.33 ) | 3.50 | 2.94 |
|  | Blood Bilirubin Increased | 88 | 2.51 ( 2.03 - 3.09 ) | 2.50 | 2.10 |
|  | Autoimmune Hepatitis | 85 | 10.3 ( 8.32 - 12.77 ) | 10.16 | 8.49 |
|  | Hepatotoxicity | 78 | 2.9 ( 2.32 - 3.62 ) | 2.89 | 2.40 |
|  | Gamma-Glutamyltransferase Increased | 77 | 2.61 ( 2.09 - 3.27 ) | 2.60 | 2.16 |
|  | Cholestasis | 73 | 3.12 ( 2.48 - 3.93 ) | 3.11 | 2.57 |
|  | Hepatocellular Injury | 72 | 3.24 ( 2.57 - 4.08 ) | 3.23 | 2.66 |
|  | Acute Hepatic Failure | 61 | 3.76 ( 2.93 - 4.84 ) | 3.75 | 3.03 |
|  | Hepatitis Cholestatic | 51 | 7.04 ( 5.34 - 9.27 ) | 6.97 | 5.54 |
|  | Hepatomegaly | 51 | 3.89 ( 2.96 - 5.13 ) | 3.88 | 3.08 |
|  | Hepatitis Acute | 47 | 5.81 ( 4.36 - 7.75 ) | 5.77 | 4.54 |
|  | Hepatic Enzyme Abnormal | 40 | 6.5 ( 4.76 - 8.88 ) | 6.45 | 4.97 |
|  | Hypertransaminasaemia | 34 | 5.15 ( 3.68 - 7.22 ) | 5.12 | 3.86 |
|  | Hepatic Cyst | 21 | 4.89 ( 3.18 - 7.51 ) | 4.86 | 3.40 |
|  | Hepatic Necrosis | 20 | 3.96 ( 2.55 - 6.15 ) | 3.94 | 2.73 |
|  | Jaundice Cholestatic | 16 | 3.5 ( 2.14 - 5.72 ) | 3.49 | 2.31 |
|  | Hepatitis Toxic | 15 | 4.4 ( 2.65 - 7.3 ) | 4.37 | 2.86 |
|  | Non-Alcoholic Steatohepatitis | 13 | 8.78 ( 5.08 - 15.18 ) | 8.68 | 5.49 |
|  | Hepatorenal Syndrome | 10 | 4.49 ( 2.41 - 8.36 ) | 4.47 | 2.65 |
|  | Non-Alcoholic Fatty Liver | 8 | 10.83 ( 5.39 - 21.78 ) | 10.68 | 5.95 |
|  | Portal Fibrosis | 6 | 22.84 ( 10.13 - 51.52 ) | 22.14 | 11.21 |
|  | Hepatorenal Failure | 4 | 5.1 ( 1.91 - 13.65 ) | 5.07 | 2.23 |
